# Incorporating patient partner scores into high stakes assessment: an observational study into opinions and attitudes

**DOI:** 10.1186/s12909-017-1063-4

**Published:** 2017-11-15

**Authors:** Fiona C. Thomson, Rhoda K. MacKenzie, Marie Anderson, Alan R. Denison, Graeme P. Currie

**Affiliations:** 1 The School of Medicine, Medical Sciences and Nutrition, University of Aberdeen, Polwarth Building, Foresterhill, Aberdeen, AB25 2ZD UK; 2 Clinic C, Aberdeen Royal Infirmary, Foresterhill, Aberdeen, AB25 2ZN UK

**Keywords:** “Patient partner”, “Volunteer or simulated patient”, “Assessment”

## Abstract

**Background:**

Volunteer patients (also known as patient partners (PPs)) play a vital role in undergraduate healthcare curricula. They frequently take part in objective structured clinical examinations (OSCE) and rate aspects of students’ performance. However, the inclusion and weighting of PP marks varies, while attitudes and opinions regarding how (and if) they should contribute towards the pass/fail outcome are uncertain.

**Methods:**

A prospective observational study was conducted to explore beliefs of PPs regarding inclusion of their scores in a high stakes undergraduate OSCE in a single UK medical school. All PPs delivering components of the local MBChB curriculum were asked to participate in the questionnaire study. Quantitative and qualitative data were analysed using descriptive statistics and framework analysis respectively.

**Results:**

Fifty out of 160 (31% response rate) PPs completed the questionnaire; 70% had participated in a final year OSCE. Thirty (60%) felt their marks should be incorporated into a student’s overall score, while 28% were uncertain. The main reasons for inclusion were recognition of the patient perspective (31%) and their ability to assess attitudes and professionalism (27%), while reasons against inclusion included lack of PP qualification/training (18%) and concerns relating to consistency (14%). The majority of PPs were uncertain what proportion of the total mark they should contribute, although many felt that 5-10% of the total score was reasonable. Most respondents (70%) felt that globally low PP scores should not result in an automatic fail and many (62%) acknowledged that prior to mark inclusion, further training was required.

**Conclusion:**

These data show that most respondents considered it reasonable to “formalise their expertise” by contributing marks in the overall assessment of students in a high stakes OSCE, although what proportion they believe this should represent was variable. Some expressed concerns that using marks towards progress decisions may alter PP response patterns. It would therefore seem reasonable to compare outcomes (i.e. pass/fail status) using historical data both incorporating and not incorporating PP marks to evaluate the effects of doing so. Further attention to existing PP training programmes is also required in order to provide clear instruction on how to globally rate students to ensure validity and consistency.

**Electronic supplementary material:**

The online version of this article (10.1186/s12909-017-1063-4) contains supplementary material, which is available to authorized users.

## Background

The involvement of simulated, standardised or volunteer patients in Objective Structured Clinical Exams (OSCEs) is commonplace in UK healthcare training programmes. They are increasingly utilised in summative assessments and widely recognised as facilitating valid and reliable assessments [1]. In our institution these people are known as patient partners (PPs). The use of PP scores in combination with clinician assessment to evaluate communication skills in medical undergraduates is now routinely accepted [2–4]. PP scores – relating to different aspects of the student encounter [5] – may be obtained in both history and examination stations, but whether these are solely used for student feedback or counted towards progress decisions through incorporation into the final student mark, and what weighting is used, varies between institutions and assessments.

There are few data exploring opinions and attitudes of PPs regarding inclusion of their mark into a student’s overall score in medical undergraduate or postgraduate high stakes exams. A small study of 14 participants exploring patient judgements of students in final year exams, found that most patients were in favour of providing student feedback but against their mark contributing towards the examination score [6]. In a further study, it was observed that standardised patients tended to rate students higher than physicians when using itemised checklists to evaluate clinical examination skills [4]. However, it is not known whether reluctance to fail underperforming students exists in PPs as has been shown by other authors [7].

The University of Aberdeen has run a volunteer patient scheme for around 15 years, initially with “simulated patients” portraying specific roles for teaching and assessing communications scenarios, and “volunteer patients” (with or without real pathological signs) for clinical examinations. The teams merged in 2013 to form the “patient partner programme” referring to the relationship between patients, staff and students in development and delivery of learning. PPs at this institution are unpaid volunteers (but receive expenses) and are largely recruited via the university website. In March 2016, a cadre of around 160 volunteers were registered with the PP programme.

For PPs involved in OSCEs, an annual training session is provided focusing on giving feedback and scoring students appropriately. Current practice across all Bachelor of Medicine and Bachelor of Surgery (MBChB) OSCEs is for PPs to independently rate students at the end of each OSCE station in response to the following:

“You should award the student up to 4 marks to reflect how well you feel you were treated, according to the following guidelines”.

**1 = Unsatisfactory, 2 = Borderline, 3 = Satisfactory, 4 = Excellent.**

“The candidate gave a clear, concise explanation and I felt able to ask him/her questions”.

**“It is very important that you are not marking the students on their medical knowledge and/or skills”.**

Wording is consistent across all exam stations and does not differ between those focusing on history and examination. PPs communicate their mark to the examiner by raising the appropriate number of fingers to prevent the student overhearing, while examiners are instructed *not* to influence the mark given by the PP. Scores are not currently incorporated into a student’s overall mark and therefore do not count towards progress decisions, but are used for feedback.

The final year MBChB OSCE in Aberdeen consists of fifteen eight minute stations, sat across two consecutive days. Students need to achieve a pass mark (set by borderline regression method) *and* pass at least ten stations. Failure to achieve this standard would result in the student having to repeat the entire year with delayed entry to the foundation programme; consequently, it is therefore considered to be a high-stakes assessment.

The aim of this study was to explore factors relating to incorporation of PP scores into the overall mark in high stakes medical undergraduate assessment (i.e. final year summative OSCE). The overarching research question was:

- What are the attitudes and opinions of PPs regarding incorporating their scores into the overall mark awarded in a high stakes assessment such as the final year medical OSCE?

## Methods

A prospective, observational study was conducted to survey the local population of PPs involved in delivering different components of the undergraduate medical (MBChB) curriculum. All PPs were invited to complete a short anonymous questionnaire (Additional file 1). Questionnaires consisted of a mixture of open, closed and free text questions. The final version of the survey was agreed upon by all authors following several changes to its design, structure and content. Questionnaires were sent by letter or email, and PPs asked to return completed questionnaires within one month; a reminder was sent two weeks after initial distribution, while the survey was also advertised within the clinical skills centre in an attempt to increase response rates. Consent to participate was implied by returning the completed questionnaire. Ethical approval for this study was granted by the University of Aberdeen College Ethics Review Board.

Questionnaire responses were collected by the PP programme staff and anonymisation ensured before passing to the main researcher for data collation and analysis using Microsoft Excel. Basic exploratory statistics were performed on quantitative data and framework applied thematic analysis of qualitative data. Transcription of free text answers into a spreadsheet, familiarisation of data and coding was performed by one of the investigators and discussed with the lead investigator to create an analytical framework of themes, that were subsequently charted and interpreted [8].

## Results

Overall, 50 out of 160 (31% response rate) PPs completed the questionnaire; demographics are shown in Table 1.

**Table 1:** Characteristics of patient partners completing the questionnaire

Gender	
Male	20 (40%)
Female	29 (58%)
Not documented	1 (2%)
Age (years)	
< 50	1 (2%)
50-59	2 (4%)
60-69	22 (44%)
70-79	22 (44%)
≥ 80	2 (4%)
Not documented	1 (2%)
Time as a patient partner (years)	
< 1	6 (12%)
1-2	4 (8%)
3-5	13 (26%)
> 5	26 (52%)
Not documented	1 (2%)
Previous participation in final year OSCE	
Never	12 (24%)
Once	10 (20%)
2-4 times	10 (20%)
5-8 times	7 (14%)
> 8 times	8 (16%)
Not documented	3 (6%)

*Q1: Do you think that Patient Partner scores should be included in a student’s overall mark in a high stakes exam (*e.g. *important degree exam such as the final year OSCE)?*

Thirty (60%) respondents felt that their marks should be incorporated into the final score in a high stakes exam, 6 (12%) felt they should not be incorporated, while 14 (28%) were uncertain.

Written responses to explain the answer to question 1 were provided by 49 (98%) respondents. Illustrative quotes in favour of or against inclusion are shown in Table 2. Common themes favouring inclusion were:

- Importance of recognising the patient perspective, *n* = 15 (31%)
- Need to assess communication skills, attitudes and professionalism, *n* = 13 (27%)
- Need to highlight and address poor interpersonal skills, *n* = 6 (12%)
- Representation of the general public, *n* = 4 (8%)

**Table 2:** Illustrative quotes favouring and against inclusion

Favouring:
*○ “In most cases body language would seem to indicate that examiner and PP scores roughly accord, but in initial patient exam followed by detailed Q + A stations, patient are often forgotten about. If, as seems likely, session times are to be extended PPs require some more input” *PP28, male aged 70-79 *○ “If they cannot treat us with dignity and professionalism at this stage it is critical that they do not progress further. More emphasis on this in year 4 should arm them with the knowledge of how vitally important the patient perspective is”* PP37, male aged 60-69 *○ “No matter how good the medical student is academically, at some point they will have to deal with the general public, ie patients. It is important that they have good ‘people skills’ therefore these skills should be tested and marked”* PP46, male aged 60-69
Against:
○ *“I do not consider myself qualified to undertake such a task”* PP15, male aged 70-79 ○ *“Many PPs have different views and would be concerned that for such an important exam our views may not be consistent”* PP29, female aged 60-69 ○ *“While I feel a good “bedside manner” is very important, I personally feel medical knowledge is of utmost importance in any consultation between patient and doctor”* PP25, female aged 60-69 ○ *“… when nerves set in candidates marks can be allotted unfairly”* PP19, male aged 60-69

Common themes against inclusion were:

- PPs not in a position to comment due to lack of qualification/training, *n* = 9 (18%)
- Concerns of consistency among PPs, *n* = 7 (14%)
- Medical knowledge more important [than interpersonal skills], *n* = 5 (10%)
- “Anxious” students inappropriately penalised, *n* = 2 (4%)

*Q2: If Patient Partner scores are included in a student’s final mark in an important degree exam, what percentage of the overall mark for each station do you think this should represent?*

**Fig. 1:**
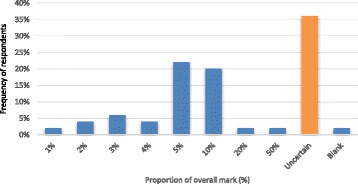
Responses to the question “what proportion of the overall score should PP marks represent?”

Most PPs (36%) were undecided what proportion this should represent, although 22% and 20% felt that 5% and 10% respectively, of the overall mark was reasonable (Fig. 1).

*Q3: If Patient Partner scores are included in a student’s final mark, do you think that further training for Patient Partners is required?*

Thirty-one (62%) felt that further training would be required if PP marks were included in the overall score, while 7 (14%) were uncertain. Common themes highlighting types of training and pre-requisites included:

- Clear criteria set for scoring, *n* = 11 (29%)
- Measures existing to ensure consistency across individuals, *n* = 9 (24%)
- Small group discussions, *n* = 7, (18%)
- Exposure to examples e.g. videos of consultations, *n* = 6 (16%)
- Detail on how scores will contribute to overall mark, *n* = 5 (13%)
- Use of experienced PPs, *n* = 4 (11%)
- Clarification that PPs assess communication [and not medical knowledge], *n* = 3 (8%)

*Q4: If a student achieves a very low Patient Partner score across the whole exam (for example, getting less than half the number of patient partner marks in total) should this result in an automatic fail of the entire exam?*

**Fig. 2:**
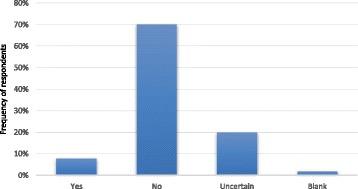
Responses to the question “if a student achieves a very low PP score across the whole exam, should this result in an automatic fail?”

The majority of PPs (35 (70%)) felt that students achieving a very low PP score across the whole exam should not automatically fail and 10 (20%) were uncertain (Fig. 2).

Forty-eight (96%) respondents provided comments with the following main themes identified:

- Examiners should decide who pass/fail, *n* = 18 (38%)
- Remedial training should be offered to students with low PP marks [regardless of overall pass/fail outcome], *n* = 14 (29%)
- Knowledge is more important [than communication], *n* = 13 (27%)
- There may be mitigating circumstances to account for low PP marks, *n* = 7 (15%)
- Students may be suited to other areas of medicine [away from patients], *n* = 4 (8%)
- Weighting of scores should preclude this eventuality, *n* = 4 (8%)

*Q5: What do you think the main advantages are if Patient Partner scores are included in the overall mark in a high stakes exam?*

Forty-four (88%) respondents provided written comments on advantages of including PP marks in the overall OSCE score. Table 3 shows illustrative quotes. Common themes included:

- Facilitate communication assessment, *n* = 18 (41%)
- Empowerment of PPs, *n* = 14 (32%)
- On behalf of the public, PPs help ensure quality of future doctors, *n* = 14 (32%)
- Students may recognise the importance of interpersonal skills, *n* = 13 (30%)

**Table 3:** Illustrative quotes – **advantages** of mark inclusion

○ *“Students would be aware that communication skills are highly regarded and are an essential part of any consultation. PPs would be more likely to feel empowered to give a mark which accurately reflects the quality of their experience with each student”* PP3, female aged 60-69
○ *“We have some input however small in a candidates attitude towards us and possibly the general public at a later date”* PP19, male aged 60-69
○ *“Students generally only value an experience that is assessed! I believe that good communication is an important aspect of being a successful doctor, therefore I think that this aspect should be included in their assessment”* PP33, female aged 60-69
○ *“In their careers, the students will have to deal with the public. Patient partners are representatives of the public during an OSCE. Therefore our views are important. Talking to patients could be considered as important as being able to take a pulse, measure blood pressure or interpret an ECG. All these skills are vital for a successful doctor therefore all these skills should be tested in an OSCE”* PP46, male aged 60-69

*Q6: What do you think the main disadvantages are if Patient Partner scores are included in the overall mark in a high stakes exam?*

Thirty-nine (76%) respondents provided written comments on disadvantages of inclusion of PP marks in the overall OSCE score. Table 4 shows illustrative quotes. Common themes included:

- Concerns regarding lack of consistency in awarding marks, *n* = 20 (51%)
- Fear of leading to inappropriate results (pass/fail), *n* = 7 (18%)
- Penalising “anxious” students, *n* = 4 (10%)

**Table 4:** Illustrative quotes – **disadvantages** of mark inclusion

○ *“As all PPs know it can be very difficult to maintain consistency of marking throughout any exam, so the pressure of knowing their mark will constitute a significant percentage of the overall mark could compound the difficulty should fatigue set in, leading to undue generosity perhaps”* PP3, female aged 60-69
○ *“If a student is nervous then perhaps he or she is unable, under exam conditions, to express themselves as they normally would. The marks of the PPs may also vary.”* PP16, female aged 60-69
○ *“Uniformity of PP attitudes, standards and life experiences cannot be guaranteed or regulated”* PP27, male aged 70-79
○ *“I suppose we all “mark” differently (some meaner than others)”* PP34, female aged 60-69
○ *“PP scores are ‘subjective’ compared with medical knowledge so there can be variation in scores given by different PPs. This could affect certain groups of patients. Any previous ‘disagreements’ or even ‘clash of personalities’ between a PP and a student that appeared during training could upset the candidate if they met In the OSCES - this could affect marks given.”* PP39, female aged 70-79

*Q7: Would you be less likely to score a student poorly if you thought this might lead them to fail the exam?*

Most respondents (32 (64%)) were comfortable in scoring a student poorly even if they thought it might lead them to fail the exam, 6 (12%) were uncertain and 8 (16%) were less likely to give low scores. Forty-six (92%) PPs provided comments explaining their answers. Those with no concerns providing a low score gave the following reasons:

- PPs should give an honest and fair opinion, *n* = 15 (47%)
- PP scores represent only one part of the whole assessment, *n* = 8 (25%)
- PPs feel a sense of duty to the University/general public, *n* = 7 (22%)

Of those less likely to give out low scores, two main reasons emerged:

- “Human nature”/“showing kindness towards candidates”, *n* = 8 (57%)
- PPs do not want the responsibility of a student failing, *n* = 4 (29%)

*Q8: The current PP score is:* ***1*** *Unsatisfactory,* ***2*** *Borderline,* ***3*** *Satisfactory,* ***4*** *Excellent.*

*Would you like to retain this scoring system?*

Approximately half (26 (52%)) of PPs considered the current 4-point scale satisfactory, while 18 (36%) felt it should be changed. Half (25) of respondents provided comments explaining their answers with 10 (20%) in favour of changing to a 5-point scoring system and 4 (8%) suggesting changes to the wording of the current 4-point system.

*Q9: Please provide any other comments you have relating to inclusion of Patient Partner scores in the overall final OSCE mark awarded to students.*

When asked to provide further comments relating to inclusion of PP scores in the overall final OSCE a further 3 themes were identified:

- Communication issues should be addressed earlier in the course, *n* = 8 (16%)
- PPs feel a sense of value from inclusion of their scores, *n* = 6 (12%)
- Fear of litigation in the case that PP marks lead a student to fail the exam, *n* = 4 (8%)

## Discussion

In the same way that examining clinicians can be viewed as experts in rating students on clinical and technical skills, patient partners can be regarded as being experts in their ability to comment on certain other aspects such as communication and professionalism. However, what is uncertain, is how PPs feel about factors surrounding the added responsibility and potential to enhance validity of the assessment by contributing towards the overall score of students in a high stakes (i.e. pass/fail) exam. This study aimed to explore this unanswered question.

Data from this experienced group of PPs (of whom 88% had spent >1 year within the local programme) have shown that most felt it was reasonable for them to contribute towards students’ overall marks in a high stakes assessment, although the majority were uncertain what proportion of the total this should comprise. Nevertheless, among PPs who expressed an opinion, 5 or 10% of the overall mark was considered acceptable. The most common reasons supporting inclusion was the notion that PPs could act as a surrogate for incorporation of a real patients perspective and that they were ideally placed to comment upon communication skills and matters of a professional nature. However, some of the reticence relating to mark inclusion included concerns relating to lack of training and standardisation/consistency across PPs. It is perhaps unsurprising that this viewpoint was expressed as at least half of the PPs at Aberdeen University have a background in education (declared on entry to the programme). However due to the anonymous nature of the survey it is not possible to comment on whether respondents to the questionnaire were representative of the entire PP group or indeed, society at large. Although 64% were comfortable in scoring a student poorly even if it was thought they might fail overall, it is important to point out that most PPs (70%) felt that in the event of uniformly low scores across an entire assessment, clinical examiners should continue to have responsibility for determining the overall outcome for such poorly performing students; a small proportion (8%) went on to express fear of potential litigation should they be held “accountable” for a student failing. Comments regarding the possibility of mitigating circumstances and need for remedial training suggest that PPs may act as advocates for students and are empathetic towards learners who fail to reach a set standard. Although most responses convey a strong message that a “poor bedside manner” is not acceptable, a significant minority tolerate progression of students with fewer interpersonal skills, expressing a belief that medical knowledge is more important than communication skills, with some suggesting employment within non-clinical areas of medicine. This dichotomy of opinion highlights the heterogeneous nature of the PP group which presumably arises from their collectively wide range of life experiences and prior contact with health professionals.

Numerous advantages of mark inclusion were offered; these primarily consisted of acknowledgement of the ability of PPs to effectively assess communication and interpersonal skills. Analysis of free-text comments suggested that recognition of these skills by the university as an institution, may foster a sense of empowerment and feelings of value amongst PPs. There was also an inference that PPs feel a sense of responsibility on behalf of the wider public, acting as representatives for health service users to ensure that a suitable standard of professionalism is reached in all graduates.

No other study has explored the views of PPs with regards to attitudes about incorporating their marks into the overall score of students, although some studies have explored relationships between scores given by PPs and those of “expert” assessors. In one cross-sectional study of 62 videotaped consultations in primary care, comparisons were made between scores given by simulated patients versus those by professional observers [2]. “Satisfaction” from the consultation by simulated patients had a predictive power of 0.74 for the observers’ assessment of trainees, while “dissatisfaction” of simulated patients had a predictive power of 0.71 for the observers’ assessment. The authors concluded that given the accordance between each group was within an “acceptable range”, simulated patient satisfaction scores could provide a reliable source for assessing communication skills in medical trainees [2]. A further study explored simulated patient and clinician evaluations of competence in an 8 station OSCE [4]. The authors demonstrated that simulated patients were as good as clinicians in providing feedback, while the former were sufficiently able to judge clinical skills. In the same study, simulated patients scored students higher than physicians (90% versus 82% respectively, *p* < 0.001) although there was only a weak correlation between simulated patients and physicians scores [4]. Another study explored the reliability of marking performed by simulated patients in a medical undergraduate OSCE and concluded that their judgements were reliable and enhanced the validity of the assessment [3]. In keeping with this, other studies have shown that patient scores can identify students with patient interaction problems that may not otherwise be recognised by clinicians, providing further opportunity for remediation of poor interpersonal skills [5, 9].

The study has some important limitations. Firstly, the response rate was 31%. Since not all PPs within this local programme participate in the final year OSCE it is possible that some felt the questionnaire was of little relevance to them. It became apparent that some PPs experienced technical difficulties with the Microsoft Word questionnaire document. To rectify this, dashed answer lines were replaced with text boxes and a second mail out occurred. No further attempts were made to increase the response rate other than a planned reminder email and advertisement of the survey on the electronic notice board within the clinical skills area. Unfortunately, some PPs may have abandoned attempts to complete the questionnaire without revisiting it once formatting issues had been resolved. In future, it would be worth considering the use of dedicated software or online survey applications to improve response rates. Nevertheless, fifty PPs did complete the questionnaire in turn providing information-rich data. Whether the respondents were representative of the total PP community and/or real patients accessing healthcare is doubtful and findings therefore need to be put into perspective, highlighting the need for further and wider research on this topic. Although PPs are “self-selected” to some extent, they are well placed to “voice” opinions relating to student performance given their experience of the OSCE format and assisting in exams.

Prior to incorporation of PP marks in “real-life” what would be required? Firstly, it would seem reasonable to compare outcomes (i.e. pass/fail status) using historical data both incorporating and not incorporating PP marks to evaluate the effects of doing so. It would also be useful to explore the opinions of other stakeholders including staff and students regarding PP mark inclusion. PP training processes should also be studied and evaluated to ensure that PP concerns about consistency in marking are addressed and clear criteria for student scoring provided. Current annual OSCE training involves watching live demonstrations of communication and clinical examination stations and advice on how to provide feedback and scores based on detailed descriptors. However, these data indicate that there is appetite for additional resources such as videoed example consultations and opportunity for small group discussions to improve calibration, while it is also suggested that experienced PPs may have a role in design and delivery of PP training. Whether or not this is a reasonable use of resources would depend on the degree of variance amongst PP scores and it would be helpful to further investigate whether perceptions regarding inconsistency and inequality translate to real discrepancies in marking. In addition PPs should be provided with appropriate information about assessment processes and how their scores may be used to add to the “wisdom of the crowd”. This may in turn reassure PPs of the value of their honest mark allocation.

## Conclusion

This study is the first to demonstrate that most PPs consider it reasonable for them to “formalise their expertise” by contributing marks in the overall assessment of students in a high stakes OSCE, although what proportion they believe this should represent was variable. Further attention to existing PP training programmes is required in order to provide clear instruction on how to globally rate students to ensure validity and consistency of the OCSE and fully evaluate the effects of mark inclusion.

## Additional file

Additional file 1: Patient Partner Questionnaire design of questionnaire agreed by all authors and distributed to the sample population for the study. (DOCX 68 kb)

## Abbreviations

MBChB: Medicine
OSCE: Objective Structured Clinical Examination
PP: Patient Partner
