# Supplementary material for: Incorporating patient partner scores into high stakes assessment: an observational study into opinions and attitudes
Source: BMC Med Educ. 2017 Nov 15;17:214. doi: 10.1186/s12909-017-1063-4 (PMC5688703; doi:10.1186/s12909-017-1063-4)
Supplement: Additional file 1: — Patient Partner Questionnaire design of questionnaire agreed by all authors and distributed to the sample population for the study. (DOCX 68 kb) [file 12909_2017_1063_MOESM1_ESM.docx]

**Patient Partner Questionnaire**

We would be grateful if you could take 10 minutes to complete the following questionnaire. It is designed to explore your opinion on the inclusion of patient partner marks into the overall mark given to medical students in important degree exams such as the final year medical school objective structured clinical examination (OSCE). All data will be anonymous and may be used to help guide inclusion of patient partner marks in future assessments.

Thank you. Please circle where appropriate:

Gender:

- Male Female Prefer not to say

Age:

- <30 30-39 40-49 50-59 60-69 70-79 >80
- Prefer not to say

How many years have you been a Patient Partner?

- < 1; 1-2 3-5 >5

Have you participated in a medical school **final year** OSCE as a Patient Partner?

- Never Once 2-4 times 5-8 times >8 times

1. Do you think that Patient Partner scores should be included in a student’s overall mark in a high stakes exam (e.g. important degree exam such as the final year OSCE)?

Yes No Uncertain

Please explain your answer:

1. If Patient Partner scores are included in students final marks in an important degree exam, what percentage of the overall mark for each station do you think this should represent? Please state a discrete number (i.e. not a range) or circle uncertain if you are not sure:

__________% Uncertain

1. If Patient Partner scores are included in students final marks do you think that further training for Patient Partners is required?

Yes No Uncertain

If yes, what sort of training would be required?

1. If a student achieves a very low Patient Partner score across the whole exam (for example, getting less than half the number of patient partner marks in total) should this result in an automatic fail of the entire exam?

Yes No Uncertain

Please explain your answer:

1. What do you think the main advantages are if Patient Partner scores are included in the overall mark in a high stakes exam?
2. What do you think the main disadvantages are if Patient Partner scores are included in the overall mark in a high stakes exam?
3. Would you be less likely to score a student poorly if you thought this might lead them to fail the exam?

Yes No Uncertain

Please explain your answer:

1. The current PP score is: **1** – Unsatisfactory, **2** – Borderline, **3** – Satisfactory, **4** – Excellent

Would you like to retain this scoring system? Yes No

If no, what would you change it to?

1. Please provide any other comments you have relating to inclusion of Patient Partner scores in the overall final OSCE mark awarded to students.

Please return your completed questionnaire by Saturday 30^th^ April 2016.
